# CorrNet: Fine-Grained Emotion Recognition for Video Watching Using Wearable Physiological Sensors

**DOI:** 10.3390/s21010052

**Published:** 2020-12-24

**Authors:** Tianyi Zhang, Abdallah El Ali, Chen Wang, Alan Hanjalic, Pablo Cesar

**Affiliations:** 1 Multimedia Computing Group, Delft University of Technology, 2600AA Delft, The Netherlands; A.Hanjalic@tudelft.nl; 2 Centrum Wiskunde & Informatica (CWI), 1098XG Amsterdam, The Netherlands; abdallah.el.ali@cwi.nl; 3 Future Media and Convergence Institute, Xinhuanet & State Key Laboratory of Media Convergence Production Technology and Systems, Xinhua News Agency, Beijing 100000, China; wangchen@news.cn

**Keywords:** emotion recognition, video, physiological signals, machine learning

## Abstract

Recognizing user emotions while they watch short-form videos anytime and anywhere is essential for facilitating video content customization and personalization. However, most works either classify a single emotion per video stimuli, or are restricted to static, desktop environments. To address this, we propose a correlation-based emotion recognition algorithm (CorrNet) to recognize the valence and arousal (V-A) of each instance (fine-grained segment of signals) using only wearable, physiological signals (e.g., electrodermal activity, heart rate). CorrNet takes advantage of features both inside each instance (intra-modality features) and between different instances for the same video stimuli (correlation-based features). We first test our approach on an indoor-desktop affect dataset (CASE), and thereafter on an outdoor-mobile affect dataset (MERCA) which we collected using a smart wristband and wearable eyetracker. Results show that for subject-independent binary classification (high-low), CorrNet yields promising recognition accuracies: 76.37% and 74.03% for V-A on CASE, and 70.29% and 68.15% for V-A on MERCA. Our findings show: (1) instance segment lengths between 1–4 s result in highest recognition accuracies (2) accuracies between laboratory-grade and wearable sensors are comparable, even under low sampling rates (≤64 Hz) (3) large amounts of neutral V-A labels, an artifact of continuous affect annotation, result in varied recognition performance.

## 1. Introduction

Emotions play an important role in users’ selection and consumption of video content [1]. Recognizing the emotions of users while they watch videos freely in indoor and outdoor environments can enable customization and personalization of video content [2,3]. Although previous work has focused on emotion recognition for video watching, they are typically restricted to static, desktop environments [1,4,5], and focus on recognizing one emotion per video stimuli [6,7,8]. For the latter case, such emotion recognition is temporally imprecise since it does not capture the time-varying nature of human emotions [9,10]: users can have and report multiple emotions while watching a single video. Here, we define fine-grained emotion recognition as recognizing the temporal moment-by-moment valence and arousal [11,12] states, typically in segments of 0.5 s to 4 s depending on the duration of an emotion [13,14]. This is in contrast to emotion recognition per video [8,15]. In this work, we draw on dimensional models of emotion (cf., Russell’s Circumplex Model of Emotions [12]), which describe emotions using a multi-dimensional space. Compared with discrete models (e.g., Self-Assessment Manikin (SAM) [16]), these have a finer level of granularity by introducing continuous variables, namely valence and arousal, to describe emotions [6].

While there has been research on fine-grained, temporally precise emotion recognition (cf., FEELtrace [17], DARMA [18], CASE [19]), these methods either require users to wear or attach obtrusive sensors [20,21,22] (e.g., Electroencephalograph (EEG)), or rely on facial expression sensing [20,21,23,24] for fine-grained emotion recognition. With respect to EEG, emotion recognition accuracies up to 80% have been achieved over the past decade [25]. However, high resolution EEG signals need to be captured under strict laboratory environments without any electromagnetic interference [26], which makes their use limited to outdoor settings. Furthermore, EEG sensors can be obtrusive since electrodes need to be attached to a user’s head during acquistion. Camera-based sensing, while less obtrusive, is not always possible in different scenarios. For example, in mobile settings, the front camera may potentially be used to unobtrusively collect facial expressions. However, the front camera cannot always capture the whole face of the user [27]. In addition, constant streaming of facial images can bring privacy concerns for both the user who watches videos and other persons whose faces may be captured in the context environment [28,29].

Unlike facial expressions, physiological signals (e.g., Heart Rate (HR), Blood Volume Pulse (BVP), Skin Temperature (ST), and Electrodermal Activity (EDA)) are largely involuntarily activated (i.e., spontaneous and not controllable), which enable a more objective means to measure affective reactions (i.e., valence and arousal) [6]. Furthermore, physiological signals can be measured using wearable sensing devices. With the proliferation of wearable physiological sensing devices (e.g., smartwatches and wristbands) that can measure signals such as HR or EDA, they have become easily accessible and widespread in daily life use [30,31]. Given the foregoing, we focus on fine-grained emotion recognition using wearable physiological sensors. To this end, we collected the Mobile Emotion Recognition with Continuous Annotation (MERCA) dataset, where users annotate their valence and arousal states using a continuous mobile annotation input technique (cf., [32]) in real-time while watching short-form videos.

Fine-grained emotion recognition needs to segment continuous signals into smaller (fine-grained) instances and recognize the emotions they represent. A major challenge for recognition is that the information inside each segment of the signals (i.e., instances) may not be sufficient for recognizing emotions. In previous works [21,33,34], sequence learning methods such as Long Short-Term Memory (LSTM) [35] networks have been used to extract the temporal information between different samples or instances as additional features for recognition. However, the temporal information extracted by sequence learning methods is based on the fine-grained emotion self-report annotated by users. Such reports may not be precise enough, be misaligned temporally to the actual state at which they were experienced, or be altogether inaccurate. If the network is trained with these labels, training error could accumulate and affect the recognition result for other instances within the same signal [36,37].

To address this challenge, this paper presents a fine-grained emotion recognition algorithm, CorrNet, which uses unsupervised learning to learn the features both inside and between different instances, and a supervised classifier to recognize the emotions for each of them. CorrNet takes advantages of the features both inside and between instances by extracting correlation-based features for all instances for the same video stimuli. Our work offers two primary contributions:

(1) We propose a novel emotion recognition algorithm to classify the valence and arousal in finer granularity using wearable physiological sensors. The proposed algorithm is tested both on an indoor-desktop dataset (CASE [19]), and on an outdoor-mobile dataset (MERCA), which we collected using wearable physiological sensors while users watched short-form (<10 min) [38] mobile videos. Results show good performance for binary valence-arousal (V-A) classification on both datasets (76.37% and 74.03% of V-A on CASE; 70.29% and 68.15% for V-A on MERCA), respectively. Our results outperform other state-of-the-art baseline methods for emotion recognition, including classic ML-based support vector machines (SVMs) and sequential learning approaches such as Long Short-Term Memory (LSTM) networks.
(2) We compare the performance of CorrNet through testing experiments with different parameters (e.g., different lengths of instances and different sampling rates) and discuss how they could affect the recognition results. The discussion provides insight into how to design a fine-grained emotion recognition algorithm using segmented physiological signals. Our discussion also shows high recognition accuracy can be achieved using wearable physiological signals with low sampling rate (≤64 Hz), which means lower power consumption and easier sensor deployment (e.g., do not need to stick electrodes on users’ skin) compared with laboratory-grade sensors with higher sampling rate (≥1000 Hz).

## 2. Related Work

In this section, we first introduce the existing models to quantify emotions. Then, we review the wearable physiological signals and existing algorithms for recognizing emotions and narrow our scope into specific techniques for recognizing fine-grained emotions.

### 2.1. Discrete vs. Dimensional Emotion Models

Emotions have been widely studied in psychology and neuroscience [39]. A variety of models have been proposed to measure and quantify emotions, which can be divided into two categories [6]: categorical and dimensional emotion models. Categorical emotion models divide emotions into different categories and describe them using emotion keywords. For example, the classic six-basic-emotion model by Ekman [40] summarized happy, sad, anger, fear, surprise, and disgust as six basic emotions, and viewed other emotions as combinations of these basic ones. Researchers also use categorical emotion models to quantify specific emotions such as frustration [41], stress [42,43], social anxiety, and depression [44]. Dimensional emotion models by contrast quantify emotions using a multi-dimensional space. Compared with categorical emotion models, dimensional emotion models can describe emotion on a finer level of granularity by using continuous values to model emotions. These models, typically Russell’s Circumplex Model of Emotions [12] which describe emotions using valence and arousal, are widely used for fine-grained or continuous emotion recognition [17,19].

Our work aims to recognize emotions in fine granularity. The emotion model we use should be able to show the dynamic and continuous changes of users’ emotion, therefore in this work we use dimensional models (i.e., valence and arousal) to model emotions.

### 2.2. Wearable Physiological Sensing for Emotion Recognition

Physiological signals collected from wearable sensors are widely used for recognizing emotions outside a laboratory environment [45,46,47,48,49]. For example, Costa et al. [45] developed an ambient intelligent system to recognize valence and arousal using Electrocardiogram (ECG), Blood Volume Pulse (BVP) and Electrodermal Activity (EDA) from iGenda, a smart wristband. Alexandros et al. [46] proposed a recognition system, HealthyOffice, to recognize stress, anxiety and depression in the workplace using ECG and BVP using a wristband and a mobile phone. Compared with signals which indicate the cognitive activities from the Central Nervous System (CNS), the signals which interpret the physiological behaviors in the Autonomic Nervous System (ANS) are easier to obtain using wearable sensors. For example, many commercialized smart watches and wristbands (e.g., Empatica E4 wristband and Toshiba W110 wristbands [50]) have integrated photoplethysmogram (PPG) and skin conductance (SC) sensors to measure Heart Rate (HR) and EDA. Recent studies have drawn on these signals to ubiquitously measure user experience, such as user engagement of mobile games [51], synchrony between presenters and audience members [49], and students’ emotional engagement during lectures [52]. However, the signals measuring signals in the ANS (normally single channel) are less information rich than Electroencephalogram (EEG) signals (normally 16–32 channels). This brings up challenges of how to design algorithms that ensure robust and accurate emotion recognition.

Our work aims to develop emotion recognition algorithms for video watching that are not limited to laboratory and indoor environments. Following prior work [45,46,49,51,52], we narrow our scope on using physiological signals such as ECG, BVP, EDA and HR from wearable sensors.

### 2.3. Emotion Recognition Algorithms Using Physiological Signals

Algorithms for recognizing emotions using physiological signals can be divided into two major categories: model specific methods and model free methods [6]. Model specific methods require carefully hand-crafted features to classify emotions from physiological signals. In general, statistical features from the time-domain (e.g., mean, standard deviation, first differential [53,54,55] of the signal) and frequency-domain (e.g., mean of amplitude, mean of absolute value [52,56], or signal FFT [51]) are commonly used. Features are selected or designed by researchers thus they do not depend on the emotion ground truth labeled by users. However, there is no consensus of which features are the most reliable for recognizing emotions [6,57]. Therefore researchers have to carefully design features according to the data they collected, limiting the generalizability of their algorithms. The extracted features are then input into classifiers such as Support Vector Machine (SVM) [58], K-Nearest Neighbor (KNN) [59], or Random Forest (RF) [60] to classify emotions. Since the model specific methods require researchers to select features based on empirical experiments, it is costly with respect to time and does not guarantee that selected features are optimized [6,7].

Model free methods on the other hand use neural networks to learn the inherent structure behind the data and automatically extract features for recognition. Deep learning networks such as convolutional neural networks (CNNs) [61,62] and Long Short-Term Memory (LSTM) networks [33,34] are commonly used and achieve high accuracy. For example, Ma et al. implemented [33] a multimodal residual LSTM network to classify valence and arousal and obtained a classification accuracy of 92.87% and 92.30% for arousal and valence, respectively. According to the research from Suhara et al. [63], LSTM networks could outperform classic machine learning algorithms such as Support Vector Machines (SVMs) for forecasting emotion states. Although model free methods achieve high recognition accuracy, they easily overfit on the training data when the ground truth labels are not accurate [64]. This appears to be a common phenomenon when users label their emotions [19,65].

Our work attempts to draw on the advantages of both model specific and model free methods by using unsupervised learning techniques to automatically extract features and supervised learning techniques to classify emotions.

### 2.4. Fine-Grained Emotion Recognition

While there exists many algorithms that are designed for recognizing emotions based on physiological signals, techniques for fine-grained emotion recognition are still in their infancy [22]. Fine-grained emotion recognition requires algorithms to output multiple emotion states by relying on signals within one certain time interval. For temporal signals, this is normally done using two kinds of methods:

The first kind of methods views the target emotion states as a continuous sequence and directly calculate the mapping (regression) from input signals to output emotion sequences. These methods include sequential learning approaches such as LSTM [33,34], and temporal regression such as support vector regression (SVR) [66,67] and polynomial regression [68]. While previous work has shown that regression approaches, especially sequential learning using recurrent structures can achieve high accuracy [10,20,69], these methods are sensitive to the accuracy of the ground truth. Since the recurrent structure is trained from the beginning to the end of the signal, the regression error from the first few samples could be accumulated and affect the results of the whole sequence.

The second kind of methods segments continuous signals into different fine-grained instances and classifies the emotion of each instance independently. Therefore, the recognition result of different instances will not affect each other. For example, Romeo et al. [70] designed an SVM-based multi-instance learning algorithm to recognize valence and arousal for each fine-grained instance and achieves 68% of accuracy on high arousal. These kinds of methods are also widely used for fine-grained emotion recognition with different data modalities such as facial expressions [71] and vocal features [72] (e.g., pitch and loudness). The main challenge for this kind of methods is to extract and fuse both the features inside and between instances, as the information which resides only within instances may not be enough to determine which emotion it represents. Previous works [70,73] use the joint loss [74] of instances and bags (instances under one video stimuli) to fuse the features inside and between instances. However, it could lead to temporal ambiguity of emotions as instances are not directly trained by their emotion labels (and instead trained by the label of bags) [70].

In our work, we draw on the second kind of methods (due to imprecision of fine-grained emotion ground truth from self-reports), and aim to extract and fuse the information within and between instances without compromising the link between instances and their emotion labels.

## 3. Methodology

**Figure 1:**
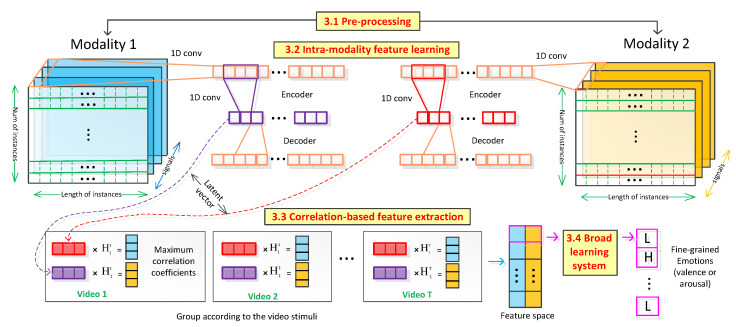
The procedure of proposed CorrNet.

In this section, a correlation-based emotion recognition algorithm (CorrNet) is proposed to classify fine-grained emotion states (i.e., valence and arousal (V-A)) from physiological signals. The procedure of the proposed algorithm is illustrated in Figure 1. CorrNet contains three stages: (1) Intra-modality feature learning: the obtained physiological signals are firstly grouped into two modalities (signals from two different nerve systems, e.g., oculomotor nerve system and autonomic nervous system). At the first stage, original signals are projected into a low dimensional latency space where intra-modal features are learned using a convolutional auto-encoder. After that, the feature vectors from the latency space are grouped according to the video stimulus the users watched. (2) Correlation-based feature extraction: In the second stage, the cross-modal features are obtained through correlation-based feature extraction. (3) Broad Learning System classification: At the last stage, the extracted features are inputted into a broad learning system (BLS) to classify valence and arousal for each instance. Each stage is discussed below, and the pseudocode of CorrNet is shown in Algorithm 1.
**Algorithm 1** CorrNet**Input:** Training set with n instances in modality 1: $X_{1}={\left\{x_{1}^{i}\right\}}_{i=1}^{n},x_{1}^{i}\in R^{L\times C_{1}}$ and modality 2: $X_{2}={\left\{x_{2}^{i}\right\}}_{i=1}^{n},x_{2}^{i}\in R^{L\times C_{2}}$ **Output:** Fine-grained emotion labels (i.e., valence:$V_{a}={\left\{v_{i}\right\}}_{i=1}^{n}$ and arousal $A_{r}={\left\{a_{i}\right\}}_{i=1}^{n}$)1:**for** j = 1 and 2 **do**2:  **Encoder**→$\phi_{j}=X_{j}⊗\psi \left(\omega ,c\right)$3:  **Decoder**→$\eta_{j}=\phi \bar{⊗}\psi^{\prime }\left(C_{j},c\right)$4:**end for**5:**Group instances according to video stimulus:**6:**for t** in **T** = number of video stimulus **do**7:  $\left(H_{1}^{t},H_{2}^{t}\right)=\mathrm{CCA}\left(\psi_{1}^{t},\psi_{2}^{t}\right)$
8:  $F^{t}=\left[\psi_{1}^{t}\cdot H_{1}^{t},\psi_{2}^{t}\cdot H_{2}^{t}\right]$
9:**end for**10:$F={\left\{F^{t}\right\}}_{t=1}^{T},F\in R^{n\times k}$11:${\left(a_{i},v_{i}\right)}_{i=1}^{n}=\mathrm{BLS}\left(F\right)$


### 3.1. Pre-Processing

Suppose $S={\left\{s_{c}\right\}}_{c=1}^{C}$ is the set of obtained physiological signals, where *C* is the number (channels) of physiological signals. The signals are firstly segmented into multiple instances with a fixed length *L*. After the segmentation, the input of the algorithm become $X={\left\{x^{i}\right\}}_{i=1}^{n}$, where $x^{i}\in R^{L\times C}$. The starting and the ending points of an instance are the starting and ending timestamps of the segmentation, respectively. The goal of CorrNet is to classify the V-A for each instance. For that, input *X* is divided into two modalities $X_{1}={\left\{x_{1}^{i}\right\}}_{i=1}^{n},x_{1}^{i}\in R^{L\times C_{1}}$, and $X_{2}={\left\{x_{2}^{i}\right\}}_{i=1}^{n},x_{2}^{i}\in R^{L\times C_{2}}$ ($C_{1}+C_{2}=C$) based on the information these physiological signals represent. For example, the two modalities could be oculomotor nerve system (ONS) and autonomic nervous system (ANS), where the signals from ONS (pupil dilation [75] and saccadic eye movement [76]) and from ANS (skin conductance [77] and skin temperature [78]) are grouped together, respectively.

### 3.2. Intra-Modality Feature Learning

The purpose of intra-modality feature learning is to (a) fuse the information from different signal channels within a modality and (b) learn local features within each instance. To achieve this target, a two-layer convolutional auto-encoder [79] is implemented. We use just shallow structure (two layers) instead of deep to avoid overfitting since each instance does not contain much information.

Suppose that $\phi_{1}={\left\{\varphi_{1}^{i}\right\}}_{i=1}^{n},\varphi_{1}^{i}\in R^{\omega }$ is the latent vector of $X_{i}$ in modality 1, where ω is the dimension of the latent space, the $\phi_{1}$ can be obtained by 1D convolution:

(1) $$\phi_{1}=X_{1}⊗\psi \left(\omega ,c\right)=X_{1}\dot{⊗}\psi_{11}\left(1,1\right)\bar{⊗}\psi_{12}\left(\omega ,c\right)$$

where $\dot{⊗}$ and $\bar{⊗}$ are the convolution operations on the dimension of channels and length of instances, respectively. $\psi_{11}\in R^{1\times C_{1}}$ and $\psi_{12}\in R^{c\times 1}$ are the convolution kernels for two layers, where *c* is the size of the convolution kernel. The first convolution layer fuses information from different channels while the second layer extracts local features between different time samples inside each instance. The latent vectors are then reconstructed using a convolutional decoder:

(2) $$\eta_{1}=\phi_{1}\bar{⊗}\psi^{\prime }\left(C_{1},c\right)$$

where $\psi^{\prime }\in R^{c\times 1}$ is the convolution kernel for the decoder. The auto-encoder-decoder is trained by minimizing the binary cross entropy [80,81]:

(3) $$H=-\frac{1}{n\cdot L}\sum_{i=1}^{n}\sum_{j=1}^{L}x_{1}^{ij}\cdot log\left(\eta_{1}^{ij}\right))+\left(1-x_{1}^{ij}\right)\cdot (1-log\left(\eta_{1}^{ij}\right)$$

where $x_{1}^{ij}$ and $\eta_{1}^{ij}$ are the *j* sample point in the instance of $x_{1}^{i}$ and $\eta_{1}^{i}$, respectively. The latent vector $\phi_{1}$ learned from the auto-encoder is the intra-modality features we want to obtain. The latent vector $\phi_{2}$ for modality 2 can be calculated using the same method.

### 3.3. Correlation-Based Feature Extraction

In this stage, intra-modality features $\phi_{1}$ and $\phi_{2}$ are fused using a correlation-based feature extraction method [82]. The purpose of correlation-based feature extraction is to extract features which (a) maximize the correlation coefficient between two modalities and (b) fuse the features between different instances. The precise classification for each instance needs to take advantage of both local information within each instance and global information between different instances, as the change of signals are sometimes not synchronized with the change of emotions. Here, we hypothesize that the same video stimuli will trigger relatively similar valence and arousal across physiological responses among different subjects. Thus, the features from instances under the same stimuli are fused with the features from the other modality by maximizing the correlation between two modalities. The transformation which maps signals to features is a weak constraint because it is a linear mapping which does not bring new linearly independent features. If we use audio-visual features (which would be the same for all subjects for one video) from video content, it will bring strong constraints to all instances for subjects watching one video. In the extreme case, the classifier could rely only on the content-based features and discard the information from physiological signals. The linear transformation however extracts features that differ across subjects, so we do not have the same features for all subjects. Here, we use linear transformation instead of other complex transformations (e.g., deep structure [83]) to lower the computational cost and avoid overfitting (where a strong constraint can make the two modalities have a correlation coefficient of ≈1).

To extract correlation-based features, we first calculate the covariance ($S_{11}$ and $S_{22}$) and cross-covariance ($S_{12}$) of the two modalities:

(4) $$S_{11}=\frac{\left(\phi_{1}^{t}\right).^{T}\phi_{1}^{t}}{D^{t}-1}+I^{\omega \times \omega },S_{12}=\frac{{\left(\phi_{2}^{t}\right)}^{T}\phi_{1}^{t}}{D^{t}-1},S_{22}=\frac{{\left(\phi_{2}^{t}\right)}^{T}\phi_{2}^{t}}{D^{t}-1}+I^{\omega \times \omega }$$

where *I* is the unit matrix and ω is the dimension of the latent space, $D^{t}$ is the dimension of $\phi_{1}^{t}$. Then, we implement the Singular Value Decomposition (SVD) on the equation below:

(5) $$\left[U,D,V\right]=\mathrm{SVD}\left(V_{1}D_{1}V_{1}^{T}\cdot S_{12}\cdot V_{2}D_{2}V_{2}^{T}\right)$$

where $D_{1}$ and $D_{2}$ are diagonal matrices whose diagonal elements are the *k* biggest non-zero eigenvalues of $S_{11}$ and $S_{22}$, respectively, where $D_{1}=\mathrm{diag}\left(\frac{1}{\sqrt{D_{11}}},\frac{1}{\sqrt{D_{12}}},\ldots ,\frac{1}{\sqrt{D_{1k}}}\right)$ and $D_{2}$ have the same format). $V_{1}=\left[V_{11},V_{12},\ldots ,V_{1k}\right]$ is composed of the *k* corresponding eigenvectors of $\left[D_{11},D_{12},\ldots ,D_{1k}\right]$, respectively, where $V_{2}$ is calculated using the same method. Now, the two linear projections $\left(H_{1}^{t},H_{2}^{t}\right)$ can be calculated by:

(6) $$H_{1}=V_{1}D_{1}V_{1}^{T}\cdot U^{\prime },\;H_{2}=V_{2}D_{2}V_{2}^{T}\cdot V^{\prime }$$

where $U^{\prime }$ and $V^{\prime }$ consist of the first *K* columns of U,V, respectively. At last, the correlation-based features of $\phi_{1}^{t}$ and $\phi_{2}^{t}$ can be obtained by: $F^{t}=\left[\phi_{1}^{t}\cdot H_{1}^{t},\phi_{2}^{t}\cdot H_{2}^{t}\right]$. We then implement the above procedure among all the *T* stimuli and get the correlation based features $F\in R^{n\times 2K}$ for all *n* instances.

### 3.4. Broad Learning System For Classification

While the previous two stages focus on unsupervised feature extraction, the last stage (Figure 1) focuses on a supervised classifier. Here, a Broad Learning System (BLS) [84] is used to map the extracted features to valence and arousal. Compared with deep learning systems such as Deep Belief Networks (DBNs) [85] and Convolutional Neural Networks (CNNs) [86], BLS is less time-consuming because it does not need to use gradient descent to train the network with multiple epochs. BLS maps the original training data into two high dimensional nodes (i.e., feature nodes and enhance nodes). Instead of using backpropagation to calculate the weights between the nodes and labels, BLS calculates the weights through pseudo-inverse, which makes the classification process faster and lowers likelihood of avoid overfitting [87].

Suppose $F^{\prime }\in R^{n^{\prime }\times 2K}$ is the training set selected from the features $F\in R^{n\times 2K}$. We first normalize $F^{\prime }$ to have mean of 0 and standard deviation of 1 using z-score normalization [88]. Then, the first feature node $A_{1}$ can be calculated by:

(7) $$A_{1}=F^{\prime \prime }\cdot W_{A_{1}}$$

where $F^{\prime \prime }=\left[F^{\prime }|1\right]$ is the augmented matrix of $F^{\prime }$. $W_{A1}\in R^{2K\times N_{1}}$ is the sparse autoencoder [89] of a random matrix $W^{\prime }$ whose element $w_{ij}^{\prime }\in \left[-1,1\right]$ are random numbers. BLS use random matrices as transformation matrices to map training data into high dimensional space. Although this method is fast, the nature of randomness suffers from unpredictability [84]. That is why an autoencoder is used to to slightly fine-tune the random nodes to a set of sparse and compact nodes. Generally, the sparse autoencoder can be obtain by solving a optimization problem [89]:

$$W_{A1}=\arg\max||W^{\prime }\cdot W_{A1}-H^{\prime \prime }{\left|\right|}_{2}^{2}+\lambda ||W_{A1}{\left|\right|}_{1}$$

(8) $$W_{A1}\cdot H^{\prime \prime }=W^{\prime }$$

where $\lambda =10^{-3}$ is the regulation parameter.

With the same method, we can generate all $N_{2}$ high-dimensional nodes $A={\left\{A_{i}\right\}}_{i=1}^{N2}$. Then, we calculate the enhance nodes *B* by:

(9) $$B=\mathrm{tansig}[\frac{A^{\prime }\cdot \mathrm{orth}\left(W^{\prime \prime }\right)\cdot S}{\max(A^{\prime }\cdot \mathrm{orth}\left(W^{\prime \prime }\right))}]$$

where $A^{\prime }=\left[A|1\right]$ is the augmented matrix of *A*. $\mathrm{orth}(W^{\prime \prime })$ stands for the ortho-normalization of the random matrix $W^{\prime \prime }$, whose element $w_{ij}^{\prime \prime }\in \left[-1,1\right]$ are random numbers. S=1200 is the shrinkage parameter of the enhanced nodes. $\mathrm{tansig}=\frac{2}{1+e^{-2t}}-1$ is the active function for the enhance nodes. After that, we can obtain the input nodes E=[A,B] in the two high dimensional spaces.

The last step of BLS is to calculate the weights between the input nodes and labels. Suppose the network can be presented as EW=y, where the *W* is the connection weights between the input nodes *E* and output labels $y,y=A_{r}$ (arousal) or $y=V_{a}$ (valence), the weights can be obtained by $W=E^{-1}y$. Although the real inverse $E^{-1}$ is hard to calculate, we can estimate *W* with pesudo-inverse [84]:

(10) $$W={\left(E^{T}\cdot E+I^{n^{\prime }\times n^{\prime }}\cdot C\right)}^{-1}E^{T}\cdot y$$

$C=2^{-30}$ is the regularization parameter for sparse regularization. After this, the network has been established and all parameters are settled. If a new sample $E_{t}$ comes, the output $y_{t}$ can be obtained by $y_{t}=E_{t}\cdot W$.

## 4. Datasets

To evaluate the performance of CorrNet, we test it on two datasets: CASE and MERCA. To the best of our knowledge, Continuously Annotated Signals of Emotion (CASE) [19] is the only published dataset which has continuously self-annotated physiological signals. However, the CASE dataset is collected in an indoor, desktop environment. To verify the validity of CorrNet using wearable physiological sensors, we collected continuous self-annotated physiological signals. Here, users annotated their valence and arousal levels using a continuous mobile annotation technique (cf., [32]) in a controlled, outdoor environment. This data collection resulted in the Mobile Emotion Recognition with Continuous Annotation (MERCA) dataset, which we describe below in Section 4.2. Testing on MERCA allows us to additionally test performance across different application scenarios (i.e., CASE: indoor-desktop video watching; MERCA: outdoor-mobile video watching). Details on each dataset are shown below.

### 4.1. CASE Dataset

**Figure 2:**
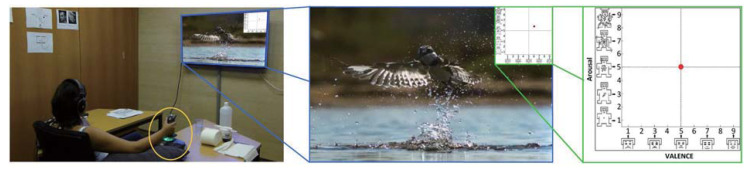
The experiment setup and annotation interface for CASE [19].

The CASE dataset [19] contains physiological recordings from 30 participants (15 m, 15 f), aged between 22–37. Valence and arousal are annotated by participants using a physical joystick (shown in Figure 2) while they watched eight video clips on a desktop screen. The data collection experiment for CASE is a 1 (task: watch videos and continuously annotate emotions) × 4 (video emotions: amusing vs. boring vs. relaxing vs. scary) within-subjects design, tested in an indoor laboratory environment. Eight video clips (two videos per emotion, duration M = 158.75 s and SD = 23.67 s) were selected to elicit the corresponding emotions. These videos are clips chosen from movies and documentaries. The emotional content of the videos used in CASE dataset was verified in a pre-study [19]. The authors first selected 20 video clips from previous works [90,91] and thereafter let 12 participants (no overlap with the participants of the data collection experiment) view and rate these videos. Then the eight videos that have the highest inter-annotator agreement were selected. Six sensors (ECG, BVP, EDA, RESP, TEMP, EMG (3 channels), shown in Table 1) were equipped to collect physiological signals. All sensors were synchronized and sampled at 1000 Hz (sample size: 2,451,650 samples × 8 signals × 30 participants). The V-A ratings (sample size: 49,033 samples × 2 annotations × 30 participants) were collected in 20 Hz according to the sampling rate of the physical joystick.

### 4.2. MERCA Dataset

#### 4.2.1. Experiment Setup

**Figure 3:**
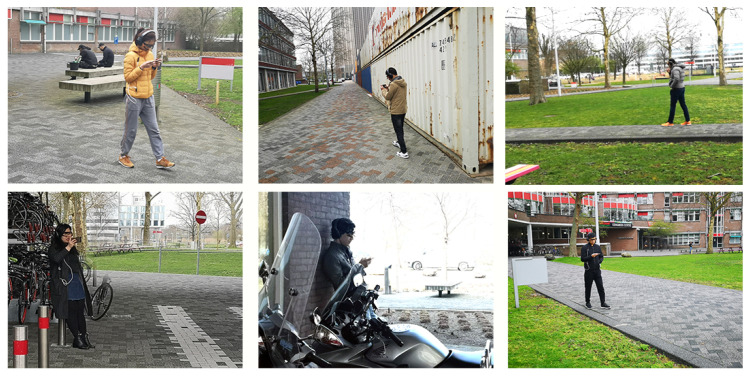
The experiment environment of MERCA. Participant photos shown with permission.

**Figure 4:**
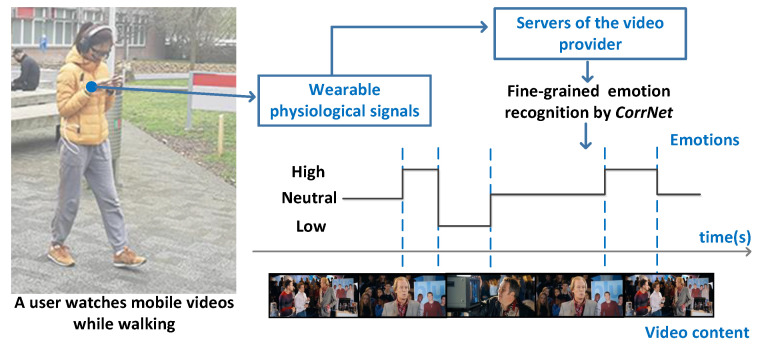
The illustration of CorrNet for evaluating mobile video watching user experience.

In total, 20 participants (12 m, 8 f) aged between 22 and 32 participated in the data collection experiment of MERCA. The number of participants in MERCA dataset is similar to some of the widely used emotion recognition datasets (e.g., CASE [19], K-EmoCon [92], DECAF [93]) with continuously annotated physiological signals. Participants were recruited from different institutions with diverse backgrounds, education levels and nationalities. All were familiar with watching videos on smartphones, and none reported visual, auditory or motor impairments. Our experiment strictly followed human data collection guidelines through our institute’s ethics and data protection committee, where informed consent was obtained from all participants. As in CASE, the data collection experiment for MERCA followed a 1 (task: watch videos and continuously annotate emotions) × 4 (video emotions: joy vs. fear vs. sad vs. neutral) within-subjects design. As shown in Figure 3, the experiment was conducted in the outdoor campus of our institute. Participants could walk or stand freely while watching videos. Participants were told to watch the videos as they normally would in such settings. To prevent participants from running into obstacles, traffic, or other people, the experimenter always accompanied the participant from a distance to guarantee their safety. The experiment setting parallels watching mobile videos while walking or waiting for a bus or train, which is a common phenomenon in mobile video consumption [94,95,96]. Figure 4 illustrates how our experiment setting parallels the application scenario of evaluating the user experience when watching mobile videos. When watching mobile videos, users would be equipped with wearable sensors to measure their physiological signals ubiquitously (with their consent). The signals will then be sent to the servers of the video provider to recognize the emotions of users in fine granularity. Lastly, the obtained emotions will be aligned with the video content for the video providers to analyze the relationship between video content and user emotions.

#### 4.2.2. Video Stimuli

In total, 12 video clips (three videos per emotion, duration M = 81.4 s and SD = 22.5 s) were selected to elicit the corresponding emotions. Ten-second black screens were added before and after each video to decrease the effects of emotions overlapping among different videos. We chose the 12 videos according to 2D emotion annotations from the self-reports in MAHNOB dataset [97]. We use the videos in MAHNOB dataset because it is a widely used dataset [98,99] with emotion self-reports from more than 30 reviewers. We selected more videos compared with CASE because we aim to collect more samples for each emotion.

#### 4.2.3. Software Setup

**Figure 5:**
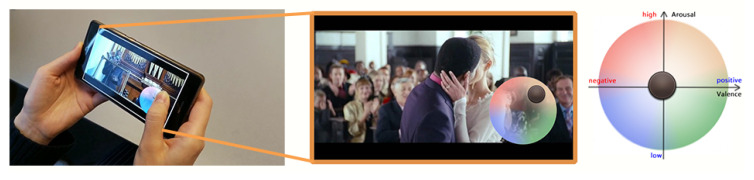
The real-time and continuous V-A annotation interface (cf., [32]) used for MERCA.

Emotions (as V-A) are annotated by participants using a real-time, continuous emotion annotation (RCEA) mobile application [32]. Participants can input their valence and arousal using a virtual joystick (shown in Figure 5) on the screen of the mobile device which they use for video watching. The virtual joystick is designed based on Russell’s Circumplex model [100]. The x and y axes of the joystick represent valence and arousal, respectively. Four colors are selected for four quadrants of the joystick base on Itten’s color system [101] to give users feedback on which emotion users are currently annotating. A gradual transparency from the origin (0% transparency) to the edge (100% transparency) of the joystick is designed to minimize the overlapping area between the video player and the virtual joystick. The transparency is also an indication of the transition of V-A intensity. We also map the frame colors to each corresponding V-A quadrant for additional peripheral feedback of which emotion users are currently annotating. Before the experiment, a 15-minute tutorial was given to familiarize participants with the operation of annotating.

#### 4.2.4. Data Collection

We used the Pupil Core wearable and Empatica E4 wristband to collect signals from Autonomic Nerve System (ANS) and Oculomotor Nerve System (ONS), respectively. We chose these two devices because they are wearable, which are suitable for collecing signals in outdoor environments and have been used by previous studies [30,102,103]. We placed the Empatica E4 tightly on users’ wrist to avoid movement of the electrodes and that was checked by the experimenter whenever the experiment started. The experimenter also checked whether the electrodes are in the right position and the recording device could get stable signals instead of noise. We waited approximately three minutes before the start of the experiment to make sure the signal collection is stable.

**Figure 6:**
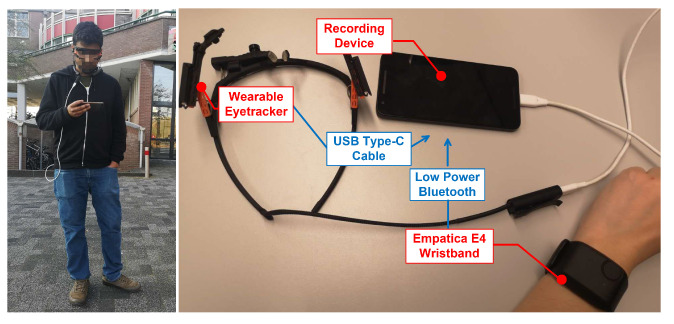
The hardware setup of MERCA. Image of study participant shown with permission.

From Empatica E4, we collected HR (1326×20) (sample size, samples × participants), BVP (84864×20), EDA (5304×20) and TEMP (5304×20) (shown in Table 1). From the wearable eyetracker, we collected pupil dilation (13260×20), saccadic amplitude (13260×20) and saccadic velocity (13260×20). Data from these two sensors were stored on one mobile device (the recording device). As shown in Figure 6, the eye tracker and E4 wristband were connected to the recording device through a USB-C cable and low-power bluetooth, respectively. The data from the two devices do not interfere with each other because they are connected to the recording device using different ports. Another mobile device (the displaying device) was used for showing the videos and collecting annotations. A noise-cancelling headphone was connected to the displaying device via Bluetooth. Timestamps of both devices were set according to the clock of the recording device, where all data is synchronized via an NTP server. The V-A ratings (sample size: 13,260 samples × 2 annotations × 20 participants) were collected in 10 Hz according to the sampling rate of the virtual joystick. The annotations on video level (post-stimuli) consist of 52.28% of all the annotations for the entire video watching. In total, 85.41% of annotations in one video watching are distributed across different VA planes, which demonstrates that different emotions can occur within one video watching.

## 5. Experiment and Results

In this section, we first introduce the implementation details of CorrNet for the CASE and MERCA datasets. We then evaluate the performance of CorrNet by both subject-dependent (SD) and subject-independent (SI) models, and compare with state-of-the-art approaches. Then, we conduct an ablation study to analyze the impact of different components in CorrNet. Lastly, we discuss about the computational complexity of the CorrNet.

### 5.1. Implementation Details

To decrease measurement bias in different trials, all signals (both CASE and MERCA) are normalized to [0,1] using Min-Max scaling normalization:

(11) $$S_{n}=\frac{S-\min(S)}{\max(S)-\min(S)}$$

Normalization is implemented on each subject under each video stimuli (trial). Since signals in MERCA have different sampling rates, they are interpreted to the 32 Hz using linear interpretation [104]. Since the sampling rates of V-A and signals are 20 and 1000 Hz respectively, we down-sampled all the signals to 50 Hz by decimation down-sampling [105] (the choice of down-sampling rates is discussed in Section 6.2). The EDA signals were first filtered using a low pass filter with a 2 Hz cutoff frequency to remove noise [106]. For the BVP signal, we pre-processed it with a four-order butterworth bandpass filter with cutoff frequencies [30, 200] Hz to eliminate the bursts [107]. An elliptic band-pass filter with cutoff frequencies [0.005, 0.1] was used to filter the ST signal [108]. We followed the standard filtering procedure widely used in previous works [6,106,107,108] to pre-process the physiological signals. Then the filtered signals are segmented into 2-second (sample size: 100 for CASE, 64 for MERCA) instances (the different choice of the segmentation length is discussed in Section 6.1). The intra-modality features are trained using adadelta optimizer [109] since it can automatically adapt learning rate. We used the Early-Stopping [110] technique to terminate training intra-modality features if there is no improvement on the validation loss for five epochs. The choice of other hyperparameters is listed in Table 2.

We set ω to L/4 = 0.5 s because 0.5 is the smallest duration of emotions [13,14]. The dimensions of latent space and output of the correlation-based features are selected based on parameter optimization. If we increase ω and *K*, the latent vector and correlation-based features will start to contain redundant information (repeated values for all latent vectors and zeros for all correlation-based features). Our model is implemented using Keras. All our experiments are performed on a desktop with NVIDIA RTX 2080Ti GPU with 16 GB RAM.

### 5.2. Evaluation Protocol and Baselines

#### 5.2.1. Classification Tasks

Three classification tasks were tested across both datatsets: (1) binary classification for low/high level of arousal and valence, (2) 3-class classification for low/neutral/high level of arousal and valence, (3) 4-class classification for the four quadrants of V-A space. We use the mean V-A of each instance as labels for classification. The mapping from continuous values of V-A to discretized categories is listed in Table 3.

#### 5.2.2. Evaluation Metrics

Three evaluation metrics are chosen to evaluate the performance of CorrNet:

- **Accuracy:** the percentage of correct predictions;
- **Confusion matrix:** the square matrix that shows the type of error in a supervised paradigm [70];
- **Weighted F1-score (W-F1):** the harmonic mean of precision and recall for each label (weighted averaged by the number of true instances for each label) [111].

These three metrics are widely used in evaluating machine learning algorithms [112]. We use weighted F1-score instead of macro and binary F1-score to take into account label imbalance.

#### 5.2.3. Evaluation Method

We train and test the proposed method using both subject-dependent (SD) and subject-independent (SI) models. Subject-dependent model was tested using 10-fold cross validation. For each subject, their data are divided into 10 folds. We train CorrNet using nine folds and tested on the remaining fold. The subject-independent model is tested using Leave-one-subject-out cross validation (LOSOCV). Data from each subject are separated as testing data and the remaining data from other subjects are used for training. The results we show are the mean accuracy and W-F1 of each fold/subject used as testing data.

#### 5.2.4. Baseline Comparison

Since there are no existing baseline methods, we compare the performance of CorrNet with both deep learning (DL) methods and classic machine learning (ML). For DL methods, we compare with 1D-CNN [113] with two and four convolutional layers. We tested 1D-CNN with a different number of convolutional layers to test whether the accuracy could be increased by making the network deeper. We also compare the performance with sequential learning approaches including LSTM [33,35] and Bidirectional LSTM (BiLSTM) [34,114] because they are widely used for the classification of time series. We train the 1D-CNN, LSTM and BiLSTM with the adadelta optimizer [109], which is the same as we used for training the intra-modality features. For ML methods, we compare with Support Vector Machine (SVM) [58], K-Nearest Neighbor (KNN) [59], Random Forest (RF) [60] and Gaussian Naive Bayes (GaussianNB) [115]. These methods are commonly used as baseline methods in datasets [62,116] and review [6,7,117] papers for affective computing. To train these ML models, we first pre-processed the signals using the same method we described in Section 5.1. We then select the mean, standard variance, average root mean square, mean of the absolute values, maximum amplitude and average amplitude for the original, first and second differential of all physiological signals. These are widely-used features for physiological signals in the task of emotion recognition [6].

### 5.3. Experiment Results

Performance of CorrNet on CASE and MERCA is shown in Table 4. In general, the subject-dependent (SD) model achieves higher accuracy and W-F1 than the subject-independent (SI) model, especially for the 3-class classification on MERCA. The accuracy of 4-class classification (four quadrants of V-A space) is lower than binary but higher than 3-class classification. Although the number of classes is higher, 4-class classification does not include testing between neutral and high/low (only two classes on V-A, respectively). Thus, the 3-class testing (high/neutral/low) on V-A independently is more challenging than four quadrants. To summarize, the overall performance on CASE is better than the performance on MERCA, which means a controlled, mobile environment can bring more challenges for emotion recognition. However, the performance on both datasets is comparable, both achieving more than 70% accuracy on binary classification and more than 60% accuracy on 3-class classification using a subject-dependent model. The results show good generalizability among different physiological signals and testing environments (desktop-indoor and mobile-outdoor).

### 5.4. Comparison with DL and ML Methods

The comparison of DL and ML methods with CorrNet using a subject-independent model is shown in Table 5. Compared with subject-dependent models, the subject-independent model is more challenging for training, which lead to less subject-bias (overfiting on specific subjects and resulting in high accuracy). Thus, we use subject-independent models to compare the performance of different methods. As shown in Table 5, for the 1D-CNN, deepening the network does not result in better performance. In fact, if we keeping increasing the number of convolution layers, the network will overfit on the training set. Here we can speculate that the information inside each instance is limited and insufficient to train a deep discriminative model. The performance of LSTM and BiLSTM is similar to 1D CNN, which means the recurrent structure does not help to increase the recognition accuracy. In general, CorrNet outperforms both ML and DL methods since it takes advantage of information across both modalities and their correlation. The only exception is that DL methods achieve higher accuracy (but lower W-F1) compared with CorrNet in 3-class classification of arousal on MERCA. High accuracy and low W-F1 means that the algorithm performs well only on a specific class (i.e., neutral arousal), which is a result of overfitting on that class. Thus, compared with DL methods, CorrNet has better performance of generalization among different classes.

### 5.5. Ablation Study

As stated, CorrNet contains three major components: intra-modality feature learning (IFL), correlation-based feature extraction (CFE), and broad learning system (BLS) for classification. We conduct an ablation study to verify the effectiveness of each component. We begin with only using the classifier on the raw signals. Then we test the performance of combining IFL and CFE with BLS independently. The results of binary classification trained using LOSOCV is shown in Table 6.

From the results, we draw the following observations: (1) Simply combining IFL and BLS does not improve classification performance when using only BLS on the raw data. IFL is a step of fusing signals from different channels and extracts local features within each instance. This is a step of information compression, thus it does not provide additional information other than what is provided from raw signals. However, it compresses the information within each instance and helps improve accuracy while combining with CFE. (2) The combination of CFE and BLS improves accuracy compared with using only BLS, however it is still lower than combining all three components. The results demonstrate the significance of fusing features between two modalities based on their correlation. (3) All components contribute to the classification task. The proposed CorrNet algorithm that jointly combines features within and between instances performs the best. These observations demonstrate the effectiveness of the proposed algorithm.

### 5.6. Computational Cost

The time complexity of CorrNet is $O\left(\left(\omega^{2}+L\omega \right)n^{2}\right)+\left(2c+1\right)\omega Ln+\omega K)$ for training and O((c+K+1)ωn) for testing. The computational cost of CorrNet is not high due to (a) the simple (2-layer) structure for intra-modality feature learning, (b) the linear mapping (instead of other complex transformation) in correlation-based feature extraction, and (c) the use of pesudo-inverse (instead of gradient descent) in broad learning. The average training time on our testing machine (desktop with NVIDIA RTX 2080Ti GPU with 16 GB RAM), is 65.56 s and 24.67 s for CASE and MERCA, respectively (sampling rate = 50 Hz). The average detection time for each fine-grained instance is 29.01 ms, which means to recognize 2 s emotions, the algorithm only spends less than 30 ms after the network is trained.

## 6. Discussion

### 6.1. Towards More Precise Emotion Recognition: How Fine-Grained Should It Be?

The length of an instance is one of the key parameters which needs to be selected carefully when designing fine-grained emotion recognition algorithms. The shorter the lengths are, the finer the granularity of an emotion that could be recognized. However, since emotion states are classified based on the information from each instance, this could entail that without sufficient information and the classification task becomes a random guess using irrelevant numbers.

**Figure 7:**
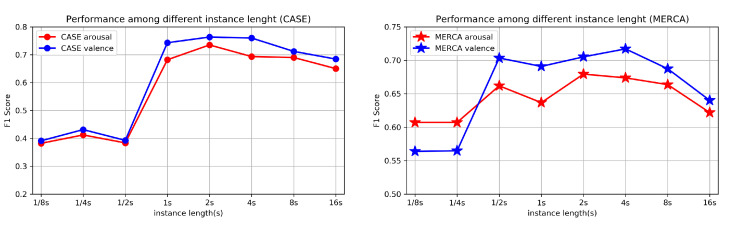
Comparison of the performance among different instance lengths: W-F1 of binary classification (LOSOCV).

To find the appropriate length of an instance, we conduct an experiment by testing CorrNet using different segmentation lengths. As shown in Figure 7, the W-F1 tested on CASE drops significantly after reducing the length to 0.5 s while the dropping threshold for MERCA is 0.25 s. This finding is in line with the finding from Paul et al. [13] that the duration of an emotion typically spans 0.5–4 s. The W-F1 also decreases after increasing the length to 8 s. Here we can speculate that overly high length instances could result in an inaccurate ground truth (more than one emotion in each instance) for classification. We find that the decrease of W-F1 on MERCA is more dramatic than the decrease on CASE, which indicates that for indoor-desktop environments, the emotion changes more slowly compared with outdoor-mobile environments (more instances with a longer length contain only one emotion). These results show that the segmentation length between 1–4 s can result in good performance (high W-F1), which can serve as an appropriate length to classify emotions using fine-grained emotion labels.

### 6.2. Emotion Recognition Using Wearable Physiological Sensing: Do Higher Sampling Rates Result in Higher Accuracies?

Traditionally, physiological sensors designed for laboratory environments often have high sampling rates (≥1000 Hz). Ideally, a higher sampling rate means better recovery of the original signal. However, a high sampling rate can also result in high power consumption and high-frequency noise, which can pose problems for usage of wearable sensors (i.e., the battery of wearable sensors is limited) in ubiquitous environments (i.e., more signal noise can occur compared with indoor laboratory environments). As our work focuses on fine-grained emotion recognition using wearable physiological sensors, it is worthwhile to investigate the influence of different sampling rates on CorrNet.

As the original sampling rate of CASE is 1000 Hz, we gradually down-sample the signals from CASE to 1 Hz and test the performance of CorrNet under different sampling rates. Although CASE was collected in a desktop environment, including it as an additional dataset helps us compare the results between laboratory-grade and wearable sensors. The down-sampling is implemented by decimating the last sampling point of every down-sampling segment. The decimate down-sampling we use is a simulation of collecting signals using wearable sensors with low sampling rate. The decimate down-sampling drops sampling points of signals in a fixed temporal interval to simulate that the A/D converter measures a continuous signal with lower frequency. Suppose the original signal $S=[s_{1},s_{2},\ldots ,s_{N}]$ and the signal after down-sampling *X* is:

(12) $$X=[x_{1M},x_{2M},\ldots ,x_{kM}]$$

where $M=\frac{F1}{F2}$, $K=\frac{N}{M}$. F1 and F2 are the sampling rates before and after down-sampling, respectively.

**Figure 8:**
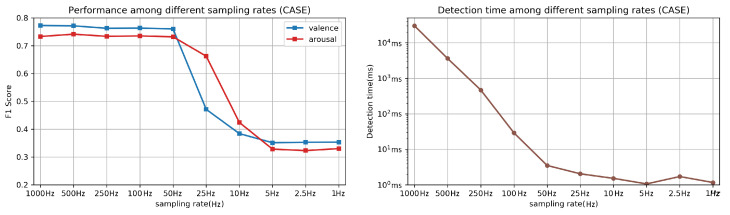
Comparison of the performance among different sampling rates: W-F1 of binary classification (LOSOCV, **left**) and detection time (**right**).

Figure 8 shows the weighed F1 score and detection time among different sampling rates. As shown in Figure 8 (left), down-sampling to 50 Hz does not significantly decrease the W-F1 score. However, the detection time for each fine-grained instance increases dramatically if we raise the sampling rate to greater than 50 Hz. This result helps explain why for most of the wearable devices (e.g., Empatica E4 wristband, BITalino Kit, the highest sampling rate of physiological sensors is less than 64 Hz (e.g., 32 Hz for Empatica E4 and 40 Hz for BITalino Kit). The comparable recognition accuracy testing on the CASE and MERCA datasets also shows low sampling rates (32 Hz) do not significantly affect the performance of emotion recognition algorithms. Our result is consistent with the findings of Martin et al. [30], where the recognition accuracy is similar between the data collected using laboratory and wearable sensors. The take away message of this experiment is that physiological signals collected from wearable devices with a low sampling rate can also be used for precise recognition of emotions (i.e., valence and arousal) for evaluating affective states during short-form video watching.

### 6.3. Data Imbalance and Overfitting in Fine-Grained Emotion Recognition

**Figure 9:**
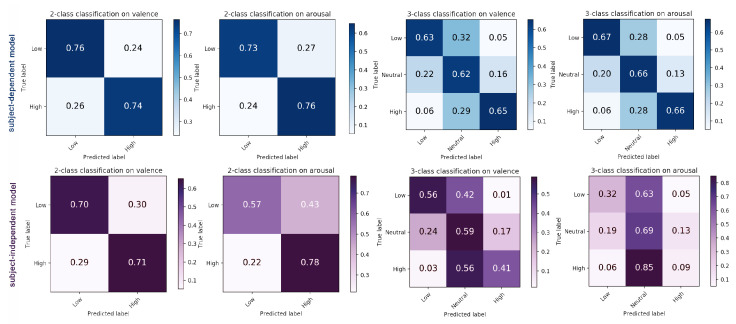
The result of 10-fold cross validation (subject-dependent model, **up**) and leave-one-subject-out cross validation (subject-independent model, **down**) on MERCA.

As shown in Figure 9 (down, LOSOCV)), there is an accuracy imbalance among different classes for 3-class classification (for binary classification we did not omit neutral labels but discrete them according to Table 3). We can see that the accuracy of class high and low (for both arousal and valence) is low, which does not occur when using the subject-dependent model. The test results on CASE are similar (instances with label of high (48%) and low (47%) are classified as neutral). Compared with the subject-independent model, the subject-dependent model is less sensitive to data imbalance, while there is still overfitting (about 30% of samples from high and low) on neutral category. We found that this can be a problem due to data imbalance when recognizing emotions using fine-grained emotion labels.

**Figure 10:**
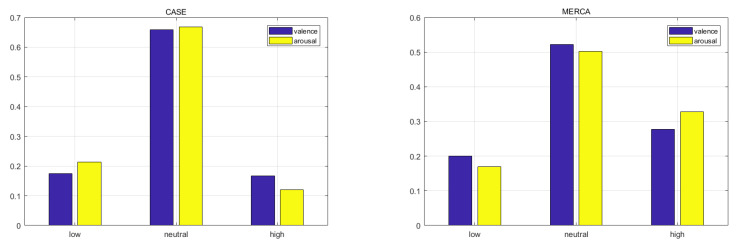
Sample percentage in each class of V-A.

As shown in Figure 10, more than 60% of samples from CASE and 50% of samples from MERCA belong to the neutral class. The resulting high amounts of neutral V-A ratings cannot be attributed to the mobile aspect of MERCA’s data collection, given that users spent most of their time (up to 73.2%) standing while watching and annotating [32]. We instead attribute this phenomenon to the act of annotating continuously, irrespective of environment (static vs. mobile). When users continuously annotate their emotions, they tend to annotate them as neutral by default (releasing virtual joystick) and non-neutral (actively annotating) only for specific scenes (e.g., kissing scenes for happy). These scenes only last for a short duration (users are not 100% of the time aroused), and for the remainder of the video clip users annotate their emotions as neutral.

The data imbalance can explain why the sequence learning techniques like LSTM do not perform well for such fine-grained emotion recognition. If most of the ground truth labels are neutral, the recurrent structure of sequence learning can easily overfit to output all classification results as neutral. The LOSOCV result shows the training accuracy of LSTM is 20.23% and 18.17% higher than the testing accuracy on CASE and MERCA respectively (averaged between V-A, 3-class classification). However, since CorrNet does not use the recurrent structure and learns the instance-label relationship independently, it does not suffer from the problem of overfitting: the training accuracy of CorrNet is only 1.01% and 4.82% higher than the testing accuracy on CASE and MERCA respectively (averaged between V-A, 3-class classification).

In addition, individuals differ in interoception levels, where self-reports of how they feel do not always correspond to their physiological response [118]. This is reflected in our observed patterns of physiological responses and continuous annotations. Thus, it also brings challenging for developing the subject-independent fine-grained emotion recognition algorithm. In general, the discussion above underscores the importance of carefully treating data imbalance and the problem of overfitting when designing any fine-grained emotion recognition algorithm.

## 7. Limitations and Future Work

Given the challenges of designing for fine-grained emotion recognition, there were naturally limitations to our work. First, although the performance of the subject-dependent model is relatively balanced among classes, the performance of the subject-independent model can still be improved if data imbalance is addressed. One promising approach is using the collected data to train a generative model (e.g., Generative Adversarial Networks [119]) to extend the size of the data for specific emotion categories (e.g., high arousal) by artificially generating more samples. Second, it is also essential for us to compare the performance of CorrNet on more datasets to further test its generalizability. However, the number of datasets with continuously annotated physiological signals is to date limited. Additionally, there are no benchmark classification results for CASE, which is the only existing dataset with continuously annotated physiological signals. Thus, it is difficult to make comparisons with more advanced learning methods. Furthermore, although the computational time is short, CorrNet was not designed to predict valence and arousal in real-time. CorrNet requires the signals (in their entirety) under one stimulus as input to extract the correlation-based features. Such prediction of emotion can help users to avoid potential negative emotions such as fatigue while driving [120], or getting distracted during lectures [52].

At last, we only consider physiological signals and do not use other modalities such as facial expressions and EEG which contain more abundant information for emotion recognition [6]. CorrNet is designed to extract the correlation-based features from signals between two modalities. Thus, it is possible to extend CorrNet to other modalities such as EEG for better recognition accuracy. In this paper, we only test it using wearable physiological signals to maximize the generalizability of it towards different potential application scenarios (e.g., mobile video watching). Facial expressions, for example, are not always possible to capture when users are on the move [27], wearing a mask [121] and Head-Mounted Display (HMD) [122], or under the conditions with inadequate light [123]. In the future, we will extend CorrNet to use signals in other modalities and investigate whether the recognition accuracy can be further improved.

## 8. Conclusions

Physiological signals from different modalities contain different aspects of human emotions. In this work, we proposed CorrNet, a fine-grained emotion recognition algorithm to classify the fine-grained valence and arousal of users using wearable physiological signals while they watch videos. CorrNet takes advantage of the information both inside each instance (segmentation of signals) and between different instances under the same video stimuli. Our algorithm achieves good performance (more than 70% of accuracy on binary classification) on two datasets that differ in setting (indoor-desktop and outdoor-mobile), and outperforms both state-of-the-art DL and classic ML methods. Our experiments on different parameters of algorithms shows fine-grained emotion recognition, typically in 1–4 s, can be achieved with high accuracy and low computational cost using wearable physiological even under low sampling rates.

## Figures and Tables

**Table 1:** Technical and physiological specifications of sensors used in CASE and MERCA dataset.

Dataset	Signals	Sensor	Sampling Rate	Physiological System
**CASE**	ECG	SA9306	1000 Hz	Autonomic Nervous System
	BVP	SA9308M	1000 Hz	
	EDA	SA9309M	1000 Hz	
	RESP	SA9311M	1000 Hz	
	TEMP	SA9310M	1000 Hz	
	EMG	SA9401M-50	1000 Hz	Facial Nerve System
**MERCA**	HR	Empatica E4	1 Hz	Autonomic Nervous System
	BVP	Empatica E4	64 Hz	
	EDA	Empatica E4	4 Hz	
	TEMP	Empatica E4	4 Hz	
	Pupil dilation	Pupil Core	10 Hz	Oculomotor Nerve System
	Saccadic amplitude	Pupil Core	10 Hz	
	Saccadic velocity	Pupil Core	10 Hz	

Electrocardiogram (ECG), Blood Volume Pulse (BVP), Electrodermal activity (EDA), Respiration (RESP), Skin Temperature (TEMP), Electromyography (EMG), Heart Rate (HR).

**Table 2:** The value of hyper-parameters in CorrNet.

Hyper-Parameter	Meaning	Value	
		CASE	MERCA
**L**	Length of each instance	2 s (100)	2 s (64)
ω	Dimension of latent space	2× L (200)	2 × L (128)
**c**	Size of conv-kernel	L/4 (25)	L/4 (16)
**K**	Dimension of corr features	L/2 (50)	L/2 (32)

**Table 3:** The mapping of V-A values and discretized classes.

Class	V-A Ratings (Binary)	V-A Ratings (3-Class)
**Low**	[1, 5)	[1, 3)
**Neutral**	-	[3, 6)
**High**	[5, 9]	[6, 9]
**4-Class**	**valence ratings**	**arousal ratings**
**High-High (HH)**	[9, 5)	[9, 5)
**High-Low (HL)**	[9, 5)	[5, 1)
**Low-Low (LL)**	[5, 1)	[5, 1)
**Low-High (LH)**	[5, 1)	[9, 5)

**Table 4:** Validation results for CASE and MERCA.

	10-Fold (SD)				LOSOCV (SI)			
	CASE		MERCA		CASE		MERCA	
	acc	f1	acc	f1	acc	f1	acc	f1
valence-2 ${}^{1}$	77.01%	0.74	75.88%	0.75	76.37%	0.76	70.29%	0.70
arousal-2 ${}^{1}$	80.11%	0.79	74.98%	0.74	74.03%	0.72	68.15%	0.67
valence-3 ${}^{2}$	61.83%	0.61	63.89%	0.63	60.15%	0.53	53.88%	0.53
arousal-3 ${}^{2}$	62.03%	0.61	66.04%	0.65	58.22%	0.55	46.21%	0.42
4-class	69.36%	0.67	72.16%	0.70	55.08%	0.53	51.51%	0.50

${}^{1}$ Binary classification. ${}^{2}$ 3-class classification.

**Table 5:** Comparison between ML, DL methods and CorrNet using LOSOCV (accuracy (W-F1)).

	**Deep Learning Methods**				
	**1D-CNN-2** ${}^{5}$	**1D-CNN-4** ${}^{6}$	**LSTM**	**BiLSTM**	**CorrNet**
**valence-2 ${}^{1}$**	58.26% (0.53)	58.00% (0.52)	48.58% (0.40)	48.81% (0.41)	**76.37% (0.76)**
**arousal-2 ${}^{1}$**	51.38% (0.44)	56.04% (0.48)	51.29% (0.38)	54.19% (0.42)	**74.03% (0.72)**
**valence-3 ${}^{2}$**	50.51% (0.38)	49.31% (0.35)	50.44% (0.35)	51.58% (0.36)	**60.15% (0.53)**
**arousal-3 ${}^{2}$**	45.89% (0.31)	47.11% (0.31)	40.52% (0.31)	42.12% (0.33)	**58.22% (0.55)**
**valence-2 ${}^{3}$**	58.13% (0.49)	56.98% (0.48)	56.01% (0.46)	59.21% (0.46)	**70.29% (0.70)**
**arousal-2 ${}^{3}$**	58.11% (0.54)	56.79% (0.53)	51.37% (0.49)	51.90% (0.50)	**68.15% (0.67)**
**valence-3 ${}^{4}$**	45.23% (0.32)	43.50% (0.32)	46.62% (0.31)	46.56% (0.31)	**53.88% (0.53)**
**arousal-3 ${}^{4}$**	45.41% (0.32)	46.56% (0.33)	**47.75% (0.32)**	47.70% (0.32)	46.21% (0.42)
	**Classic Machine Learning Methods**				
	**SVM**	**KNN**	**RF**	**GaussianNB**	**CorrNet**
**valence-2 ${}^{1}$**	49.02% (0.42)	50.76% (0.50)	48.83% (0.48)	50.99% (0.39)	**76.37% (0.76)**
**arousal-2 ${}^{1}$**	51.22% (0.42)	51.13% (0.51)	50.46% (0.49)	52.08% (0.41)	**74.03% (0.72)**
**valence-3 ${}^{2}$**	42.52% (0.30)	38.95% (0.37)	37.62% (0.35)	43.26% (0.31)	**60.15% (0.53)**
**arousal-3 ${}^{2}$**	50.18% (0.35)	43.38% (0.40)	42.29% (0.39)	27.98% (0.15)	**58.22% (0.55)**
**valence-2 ${}^{3}$**	50.92% (0.39)	51.27% (0.51)	50.78% (0.50)	48.34% (0.38)	**70.29% (0.70)**
**arousal-2 ${}^{3}$**	57.16% (0.45)	51.34% (0.51)	49.85% (0.49)	52.59% (0.42)	**68.15% (0.67)**
**valence-3 ${}^{4}$**	44.89% (0.30)	37.89% (0.36)	38.48% (0.37)	24.91% (0.15)	**53.88% (0.53)**
**arousal-3 ${}^{4}$**	44.49% (0.32)	37.52% (0.37)	38.44% (0.37)	34.68% (0.24)	**46.21% (0.42)**

${}^{1}$ Binary classification on CASE. ${}^{2}$ 3-class classification on CASE. ${}^{3}$ Binary classification on MERCA. ${}^{4}$ 3-class classification on MERCA. ${}^{5}$ 1D-CNN with 2 convolutional layers. ${}^{6}$ 1D-CNN with 4 convolutional layers.

**Table 6:** Ablation study of different components in CorrNet (accuracy (W-F1)).

	CASE		MERCA	
	Valence	Arousal	Valence	Arousal
BLS	52.68% (0.50)	56.53% (0.56)	57.26% (0.57)	57.88% (0.49)
IFL + BLS	53.79% (0.46)	57.80% (0.57)	57.96% (0.56)	58.78% (0.45)
CFE + BLS	69.80% (0.68)	66.41% (0.63)	65.43% (0.65)	63.82% (0.63)
IFL + CFE + BLS	**76.37% (0.76)**	**74.03% (0.72)**	**70.29% (0.70)**	**68.15% (0.67)**

## Data Availability

The data presented in this study are available on request from the corresponding author. The data are not publicly available due to the privacy of the participants.
